# Molecular data and ploidal levels indicate several putative allopolyploidization events in the genus Potentilla (Rosaceae)

**DOI:** 10.1371/currents.RRN1237

**Published:** 2011-05-16

**Authors:** Mats Töpel, Magnus Lundberg, Torsten Eriksson, Bente Eriksen

**Affiliations:** ^*^University of Gothenburg, Department of Plant and Environmental Sciences, P.O. Box 461, 405 30 Gothenburg; ^†^Department of Botany, Stockholm University, SE-10691, Stockholm, Sweden and ^‡^Department of Biodiversity Informatics, Swedish Museum of Natural History, Box 50007, SE-104 05 Stockholm, Sweden

## Abstract

Several naturally occurring hybrids in Potentilla (Rosaceae) have been reported, but no molecular evidence has so far been available to test these hypotheses of hybridization. We have compared a nuclear and a chloroplast gene tree to identify topological incongruences that may indicate hybridization events in the genus. Furthermore, the monophyly and phylogenetic position of the proposed segregated genera Argentina, Ivesia and Horkelia have been tested. The systematic signal from the two morphological characters, style- and anther shape, has also been investigated by ancestral state reconstruction, to elucidate how well these characters concur with the results of the molecular phylogenies.

Six major clades, Anserina, Alba, Fragarioides, Reptans, ivesioid and Argentea, have been identified within genus Potentilla. Horkelia, Ivesia and Horkeliella (the ivesioid clade), form a monophyletic group nested within Potentilla. Furthermore, the origin of the proposed segregated genus Argentina (the Anserina clade) is uncertain but not in conflict with a new generic status of the group. We also found style morphology to be an informative character that reflects the phylogenetic relationships within Potentilla. Five well-supported incongruences were found between the nuclear and the chloroplast phylogenies, and three of these involved polyploid taxa. However, further investigations, using low copy molecular markers, are required to infer the phylogeny of these species and to test the hypothesis of hybrid origin.

## Introduction

 The position of genus *Potentilla* L. (Rosaceae) in the tribe Potentilleae, subfamily Rosoideae, has been corroborated by both morphological and molecular data [1] [2] [3]. Wolf [4] presented the most extensive taxonomic work to date on the infra-generic relationships in the genus. He recognized 305 species as well as numerous naturally occurring hybrids and based his observations on morphological data. He divided the genus into six subsections based on the shape of the style and its position on the ovary and named them Rhopalostylae, Nematostylae, Closterostylae, Conostylae, Gomphostylae and Leptostylae (Fig. 1). More recent studies [5] [6] [7] have added to the inter-generic classification of the tribe Potentilleae, but little has so far been published on phylogenetic relationships within *Potentilla* [2] [3] [8] [9] [10].

**Figure:**
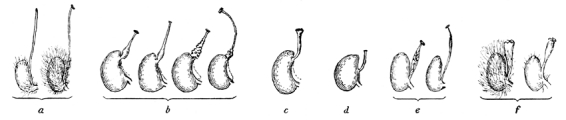
**Figure 1.** Style types in *Potentilla* recognized by Wolf (1908). (a) the long and threadlike Nematostylae, (b) the cone-shaped Conostylae, (c) the club-shaped Gomphostylae and (d) the short threadlike Leptostylae. (e) the Closterostylae type is now found in *Drymocallis* and (f) Rhopalostylae is found in the two genera *Dasiphora* and *Sibbaldianthe*. Modified from Wolf [4].

Recent systematic treatments of *Potentilla* using molecular data [2] [3] have resulted in a new circumscription of the genus. Many of the taxa that had previously been recognized by Wolf [4] as part of *Potentilla* were shown to belong to the sister clade Fragariinae. As a consequence, all species that were part of subsection Rhopalostylae (Fig. 1f) were transferred to the genera *Dasiphora* Raf. and *Sibbaldianthe* Juz. Likewise, subsection Closterostylae (Fig. 1e) is comprised of species now recognized in genus *Drymocallis*. Hence, it seems that style characters are relevant for taxonomy in Potentilleae. Furthermore, three segregated genera *Ivesia* Torr. & A. Gray, *Horkelia* Cham. & Schltdl. and *Duchesnea* Sm. were shown to be nested in *Potentilla*. Eriksson et al. [2] [3] also showed that the *Potentilla anserina* clade has an ambiguous phylogenetic position. However, a limited number of species or markers were included in these studies. Therefore, many questions about the phylogenetic relationships in the genus has not yet been answered.

Hybridization between two species will most often result in sterile offspring. Occasionally, the chromosome number of the offspring may be doubled and this can result in a fertile polyploid individual. Genome duplication within a species is termed autopolyploidization, and interspecific hybridization followed by genome duplication is called allopolyploidization [11] [12]. This classification is not entirely discriminating and consequently debated by some authors [13]. Allopolyploid speciation is suggested to have occurred several times in *Potentilla,* as many plants express morphologies that have been interpreted as intermediate between species [14] [15], and that ploidal level varies within and among species [16] [17]. However, these hypotheses of hybridization have never been tested with molecular data. Furthermore, the view that a hybrid will display characters intermediate between the parental species has been challenged by Rieseberg and Ellstrand [18]. They presented several examples where hybridization did not result in an offspring with intermediate morphology, but rather a mosaic of parental characters or new character combinations not found in the parental species. Molecular evidence is therefore important for the identification of hybrids.

A simple method for detecting hybridization is to compare gene trees from different genomes, in particular genomes with different inheritance such as plastid versus nuclear genomes. Gene tree incongruences are then considered evidence of hybridization, or indications of hybridization events (e.g. [3] [19] [20] [21] [and references therein]). It is worth noting that the two nuclear ribosomal markers ITS and ETS, used in this study, occur in many copies in the plant genome and evolve under concerted evolution [22]. This process will homogenize the repeated DNA copies towards the maternal or paternal genotype after hybridization between different species. If this homogenization acts towards the maternal genotype, no incongruence will appear between the two phylogenies, and the hybridization event will go undetected.

 As hybridization has been considered common in flowering plants, and especially within Potentilleae, such incongruences are perhaps too readily interpreted as hybridization. Other possibilities may account for incongruences, including incomplete lineage-sorting [23] [24], introgression leading to chloroplast capture [21], and unrecognized paralogy problems through gene duplication or molecular convergence. Molecular convergence in adaptive traits has been detected among animals [25] [26] [27] and are assumed to have arisen through selection acting on functional genes. Among plants, there seem to be no such examples known at the level of DNA sequence data. For example, none seem to be evident in the *rbc* *L* gene [28]. In the present study, we use intron and spacer DNA sequence data. Although there may be structural constraints (such as the maintenance of stem regions in nuclear ribosomal ITS) that would make evolution of these regions less than completely neutral, we consider molecular convergence to be a much less likely cause of conflict between topologies than hybridization. 

 It is a complex task to distinguish hybridization from incomplete lineage sorting and several methods have been suggested (e.g. [23] [24] [29] [30] [31] [32]). However, these methods are impractical in the face of our study which covers a large set of species, and because of this we have been unable to distinguish incongruences caused by putative hybridization from incongruences caused by incomplete lineage sorting. Still, in cases where differences in ploidal levels are observed, it seems reasonable that hybridization (allopolyploidy) might be the favoured explanation.

This study is a first step towards a full phylogeny of *Potentilla* with the aim to (1) study phylogenetic relationships within the genus and identify major clades, (2) evaluate the monophyly and phylogenetic position of the segregated genera *Argentina *as well as* Ivesia,* *Horkelia* and associated genera, (3) identify species or clades of putative hybrid origin, based on incongruences between chloroplast and nuclear topologies and (4) by ancestral state reconstruction investigate if two conspicuous flower characters provide systematic information that accords with the phylogenetic results. 

## Materials and Methods

*Plant material* – We have extended the dataset of *Potentilla* species used in Eriksson et al. [2] [3] in order to make a more detailed study of the phylogenetic relationships within the genus and to identify major clades of related taxa. Our analysis includes 64 ingroup taxa and seven outgroup taxa from various genera in the sister clade Fragariinae. In this study, the taxonomy is based on Atlas Flora Europeae [16] for the European species and the International Plant Name Index [33] for the non-European species. The aim has been to sample as many of the major groups proposed by Wolf [4] as possible. In addition, species belonging to the segregate genera *Ivesia*, *Horkelia*, *Horkeliella* Rydb. and *Comarella* Rydb. (the latter a section in *Ivesia* in recent treatments [34]) have been included in the analysis. Attempts were also made to include *Ivesia arizonica* (Eastw. ex J.T.Howell) Ertter (first described as *Purpusia saxosa* Brandegee) and *Ivesia santolinoides* A. Gray (later transferred to *Stellariopsis santalinoides* (A.Gray) Rydb.) but this failed due to a lack of good material to extract DNA from. In total, eight representatives from these segregated North American genera were included. Material was collected in botanical gardens, from herbarium specimens or from natural populations (Table S1). Only seeds were available for some of the included taxa, and these were sown in the spring of 2006, and leaf material was collected for DNA extraction. Vouchers were prepared when plants were in flower (stored at GB herbarium, Gothenburg).

*Morphological study* – The results from previous studies imply that the style characters used by Wolf [4] may have taxonomic value [2] [3]. Anther shape (Fig. 2) is also a character that has been proposed to carry information about species relationships, and has been used to subdivide Potentilleae [5]. Here we are interested in evaluating their usefulness within *Potentilla* as currently circumscribed by performing an ancestral state reconstruction analysis. The character states were determined by examining herbarium specimens and are summarized in Table S1. Ancestral character states were estimated by fitting three different models of rate variation, using maximum likelihood optimization and the nuclear data phylogeny. A likelihood-ratio test was used to choose which of the three models to apply. The first model assumes equal transition rates between all states (ER). In the second model, each pair of states can have a distinct rate of interchange, but this rate apply to both the forward and reverse transformation (SYM). The third model allows each pair of states to have distinct rates for both the forward and reverse transformation (ARD). The nuclear phylogeny was first converted to a fully dichotomous tree by adding zero length branches in the unresolved clades using the default setting of the function multi2di, and the analyses where then performed using the function ace in the package ape [35] of the statistical program R [36].

**Figure:**
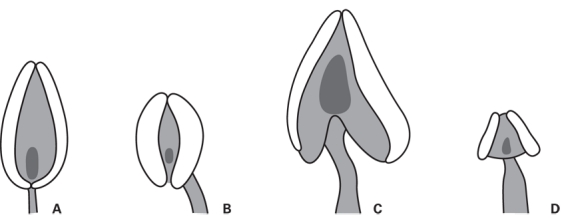
**Figure 2.** Anther shapes in *Potentilla* of the Potentillinae-type sensu Soják [5] with two thecae. A. Large, narrow anthers; connective prominent relative to thecae and entire at base. B. Medium-sized anthers; connective small relative to thecae. C. Large anthers; connective prominent and ± cordate. D. Small anthers; connective prominent, ± triangular. Letters A-D corresponds to the character states reported in Table S1.

*Chromosome counts* – Roots were pretreated in a 1:1 mixture of 0.3% Colchicin and 2mM 8-hydroxy-chinolin for 2 h at 15–20°C and transferred to Carnoy I (Acetic Acid and 95% Alcohol, 1:3) for fixation. The tissue was hydrolysed in a 1:1 mixture of concentrated Hydrochloric Acid and 95% Alcohol at room temperature for 5–10 min, washed twice with water, and stained in Aceto-Orcein for 0.5–1 h. The actively dividing root tip was dissected on a microscope slide and the cell mass squashed under a cover glass. To make slides permanent, the cover glass was removed after freezing in liquid Nitrogen. The tissue was washed and dehydrated in a series of 70%, 95% and absolute Alcohol. Before embedding in mountant the tissue was soaked in Histoclear for 24 h.

*Extraction* – Leaf material from wild or cultivated specimens was dried in silica gel before extraction. In some cases silica dried material was unavailable and herbarium material was used. For the majority of the samples, total genomic DNA was extracted using the DNeasy Plant Mini Kit (Qiagen, Valencia, California, USA). Standard protocol for plant tissue extractions was used. A minor part of the samples were extracted using the E.Z.N.A SP Plant DNA Miniprep kit (OMEGA Bio-Tec, Doraville, Georgia, USA) according to the enclosed protocol.

*PCR-amplification* – For the ITS and *trnS/G* regions we used PuReTaq Ready-To-Go PCR Beads [37]. Each reaction contained 1µl [20 µM] 5'-primer (forward) and 1µl [20 µM] 3'-primer (reverse), 0,5-4µl (typically 2µl) template DNA of unknown concentration and ultra purified water to a final volume of 25µl. Amplification of the ETS and *trnL/F* regions were performed using 25 µl MasterAmp 2x PCR PreMix G [38], 1,5µl of [20 µM] forward and reverse primers, 0,2µl Termoprime Plus DNA Polymerase [39], 0,5-4µl (typically 1µl) template DNA and ultra purified water to a final volume of 50µl. For a number of taxa extracted from herbarium material the MasterAmp 2x PCR PreMix G protocol produced low or no product at all. In these cases the PuReTaq Ready-To-Go PCR Bead protocol was used instead. PCR-products were purified with the QIAquick PCR Purification kit from QIAGEN, according to the enclosed protocol.

*Sequencing* – Dye terminator cycle sequencing with DTCS-Quick start kit (GenomeLab) were performed using a Beckman Coulter CEQ 8000 Genetic Analysis system (software v. 8.0) automated sequencer according to the manufacturer’s protocol.

*Primers used for PCR amplification and sequencing* - ETS1 and IGS6 [40] for the ETS region; ITS-1 [41], ITS2 and ITS4 [42] and ITS3B [43] for the ITS region; trn-C, trn-D, trn-E and trn-F [44] for a *trnL* intron and the adjacent *trnL-F* spacer; trnS^GCU, 3´trnG^UUC, 5´trnG2G and 5´trnG2S [45] for a terminal intron in the *trnG* region and the spacer between *trnS* and *trnG*. All regions were sequenced in both directions using both the terminal PCR primers and two internal primers (except for the ETS region where no internal primers where used).

*Sequence preparation* - Sequences were assembled and manually edited using the Staden package version 1.6.0 [46], using phred v.0.020425.c [47] for base calling and phrap v.0.990329 [48] for assembling contigs. Sequences were aligned with the software Muscle v. 3.6 [49] using the default settings followed by some additional manual editing in SeaView 4.2 [50]. Sequences from the chloroplast intergenic spacers *trnL-trnF* and *trnS-trnG*, were truncated in conserved regions of the 5' and 3' ends of the sequences and concatenated into a single matrix. Parts of the aligned matrix (corresponding to position 599-671 in sequence FN594698 from *P. thuringiaca*) around the internal primers were excluded due to sequencing problems of this region. The nuclear markers ITS and ETS were concatenated into a single matrix and truncated in both ends of the respective regions. Indels in both matrices were coded using SeqState v.1.32 [51] using the simple coding strategy [52].

*Test for recombination* – The combined ETS and ITS dataset was analyzed for indications of recombination with the following methods: RDP [53]; Bootscan/Recscan [54]; Geneconv [55]; MaxChi [56]; Chimera [57]; SiScan [58] and 3Seq [59], implemented in the RDP3 program v.3.34 [60]. Cut off of P-values where set to 0.05 with a Bonferroni correction, otherwise using the default settings. Putative recombination events that were detected by two or more methods were further investigated with the Bootscan method [54], using 100 bootstrap samples and a window size of 100-300 positions.

*Phylogenetic analysis - *Both datasets were analyzed with MrBayes v.3.2 (source code accessed with cvs 22 January 2009) compiled to use the MPI parallel library [61] [62]. Two independent analyses where run for 2 000 000 generations (nuclear data set) or 5 000 000 generations (chloroplast data set), with eight chains each and the Temp parameter set to 0.1. Trees were sampled every 1000 generations. The first 25% of the sampled trees were discarded and the remaining samples were summarized in a 50% majority-rule consensus tree. Both the nuclear and the chloroplast matrices were analyzed under the general time-reversible model (GTR) assuming a gamma shaped distribution of rate variation. MrAic.pl v1.4.3 [63] in conjunction with PHYML v.2.4.4 [64] was used to select the model. All three information criterions implemented in MrAic (AIC, AICc and BIC) rated this evolutionary model the highest. A binary model was used for the coded gaps. The mixing behavior of the mcmc chains was analyzed in Tracer v1.5 [65] and was found to have been sufficient. Mixing between the different metropolis coupled chains were analyzed in the statistics package R [36] by plotting selected columns in the *.mcmc file. In addition, two more analyses were performed based on the two matrices using the same method, but excluding the gap codes (data not shown). 

## Results

*Test for recombination –* One putative recombination event was found in the original combined ETS-ITS dataset. It involved *P. clusiana* as the daughter sequence and *P. nitida* as the major parent (the minor parent was not detected but *P. thurberi* was indicated as a candidate). The *P. clusiana* sequences were therefore excluded from both datasets. 

*Chromosome counts* – The results of the chromosome counts are presented in Table S1.

*Phylogenetic analysis *- Standard deviation of split frequencies (StdDev) were below 0.01 by the end of the two analyses (as recommended by the MrBayes manual). By changing the temperature variable in MrBayes to 0.1 from the default 0.2, the mixing between chains was dramatically improved and the analyses were assumed to have converged.

*Phylogeny* - Six major clades were identified in both the chloroplast (Fig. 3) and nuclear (Fig. 4) phylogenies. These clades were named the Anserina-, Alba-, Fragarioides-, Reptans-, ivesioid- and Argentea clades and are treated separately in the following text. Two additional analyses of the nuclear and chloroplast data sets were also performed, but excluding the indel data (results not shown). The resulting phylogenies showed only minor changes in topology, and support values were comparable to those in figures 3 and 4.

**Figure:**
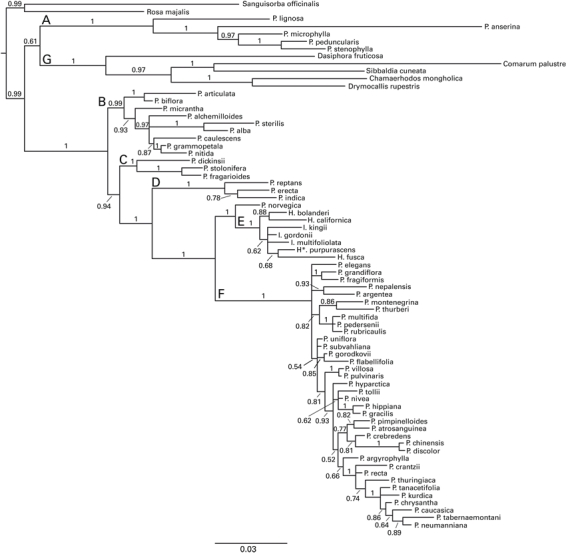
**Figure 3.** Bayesian 50% majority-rule consensus tree from the analysis of the nuclear ribosomal ITS and ETS regions from 64 species of *Potentilla* and six outgroup taxa. Six clades discussed in the text are indicated by capital letters in the tree: (A) The Anserina clade, (B) the Alba clade, (C) the Fragarioides clade, (D) the Reptans clade, (E) the ivesioid clade with *P. norvegica* as sister taxon and (F) the Argentea clade. Also indicated is (G) the Fragariinae clade that is part of the outgroup. The scale bar shows the number of inferred changes per site. Posterior probability values for clades are indicated on the branches. Species names have been abbreviated as follows: P, *Potentilla*; H, *Horkelia*; I, *Ivesia* and H*, *Horkeliella*.

**Figure:**
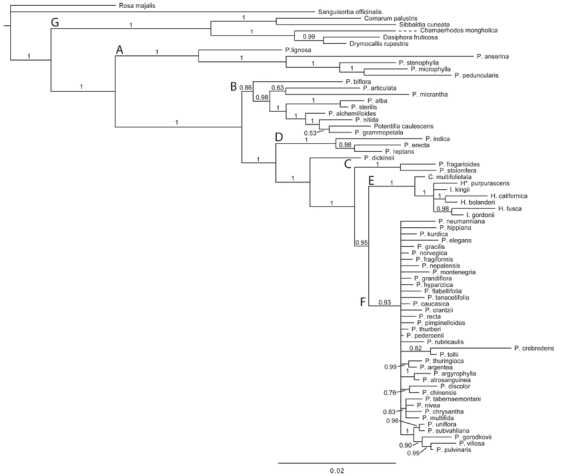
**Figure 4.** Bayesian 50% majority-rule consensus tree from the analysis of chloroplast *trnL/F*- and *trnS/G*-spacers in 64 species of *Potentilla* and six outgroup taxa. The analysis includes the same species as the nuclear analysis but the topology of the two trees are not fully congruent. (A) The Anserina clade has a well supported sister position to the rest of *Potentilla*. (C) The Fragarioides clade has shifted position with (D) the Reptans clade. *Potentilla norvegica* is not sister to (E) the ivesioid clade, but instead part of (F) the largely unresolved Argentea clade. The scale bar shows the number of inferred changes per site. Posterior probability values for clades are indicated on the branches. Species names have been abbreviated as follows: P, *Potentilla*; H, *Horkelia*; I, *Ivesia* and H*, *Horkeliella*.

The Anserina clade - *Potentilla anserina* L.*, P. microphylla* D. Don.*, P. peduncularis* D. Don.and* P. stenophylla* Diels and *P. lignosa *Willd. ex Schlecht. (clade A in Fig. 3 & 4)*. *The Anserina clade was resolved as sister, with a posterior probability (pp.) support of 1, to the rest of *Potentilla *in the chloroplast phylogeny but in the nuclear analysis weakly supported as sister to the outgroup Fragariinae (pp. 0.61). The support for a sister relationship to Fragariinae increased when the indel data were excluded from the analysis (pp. 0.84, data not shown). Divergence between *P. lignosa *and the rest of the species in the clade represented the oldest split in both phylogenies. *Potentilla stenophylla* was sister to *P. peduncularis *in the nuclear analysis*,* but *P. microphylla* had this position in the chloroplast analysis. The Leptostylae type of styles (Fig. 1d) dominated the clade and only *P. lignosa* differed by having a Nematostylae type of style (Fig. 1a).

The Alba clade - *Potentilla articulata* Franch., *P. biflora* Willd. Ex Schlecht., *P. micrantha* Ramond ex DC*.*, *P. sterilis* Garcke, *P. alba* L.,* P. alchemilloides* Lapeyr., *P. caulescens* L., *P. grammopetala* Moretti, *P. *nitida L (clade B in Fig. 3 & 4). This clade had strong phylogenetic support in the nuclear analysis (pp 0.99), but weaker support in the chloroplast phylogeny (pp. 0.86)*. Potentilla biflora* and *P. articulata* form a clade in the nuclear analysis (pp. 1) but not in the chloroplast analysis. All taxa have a Nematostylae type of style (Fig. 1a).

The Fragarioides clade - *Potentilla dickinsii* Franch. & Sav., *P. stolonifera* ex Lehm.*, P. fragarioides* L. (clade C in Fig. 3 & 4). This clade had strong support in the nuclear analysis. A clade containing *P. fragarioides *and *P. stolonifera* was also supported in the chloroplast analysis. The latter clade is sister to a clade including the ivesioid and the Argentea clade, but excluding the Alba clade in both analyses. The nuclear analyses also includes the Reptans clade as a sister clade. *Potentilla dickinsii* differed from the other two species by having a Nematostylae style rather than a Gomphostylae type of style (Fig. 1).

 Reptans clade - *Potentilla reptans* L., *P. erecta* Hampe. and *P. indica* (Andrews) T. Wolf (clade D in Fig. 3 & 4). This clade had strong support in both phylogenies, but its position relative the Fragarioides clade differs in the two trees. It is sister to a clade including the ivesioid and the Argentea clade (clades E + F) in the nuclear analysis. Its sister group in the chloroplast analysis also includes the taxa in the Fragarioides clade. Only Gomphostylae type styles were found in this clade (Fig. 1c).

The ivesioid clade* - Horkelia bolanderi* A. Gray, *H. californica* Cham. & Schltdl., *Ivesia multifoliolata* (Torr.) Keck., *Ivesia kingii* S. Watson, *Ivesia gordonii* (Hook.) Torr. & A. Gray, *Horkeliella purpurascens* Rydb. and *Horkelia fusca* Lindl (clade E in Fig. 3 & 4). This clade contained all *Ivesia*, *Horkelia* and *Horkeliella* species and had strong support in both phylogenies (pp. 1), and was sister to the Argentea clade in both the nuclear (pp. 1) and chloroplast (pp. 0.95) phylogenies. *Potentilla norvegica *L*.* was sister to the clade in the nuclear analysis, but included in the more or less unresolved Argentea clade in the chloroplast analysis. Resolution within the ivesioid clade was low, but *H. californica* and *H. bolanderi* were sisters in both phylogenies, although with low support (pp. 0.88) in the nuclear analysis. The styles were always thickened at the base (Conostylae type, Fig. 1b) like in the sister taxon *P. norvegica.* Occasionally, the styles were conspicuously elongated in species with deep hypanthia.

The Argentea clade* - *All remaining *Potentilla* taxa in this study belong to the Argentea clade (clade F in Fig. 3 & 4). This clade had a posterior probability support of 1 in both the nuclear- and the chloroplast analyses, but little internal resolution. Nevertheless, the analyses revealed seven subclades with posterior probability >0.95 in the nuclear analysis (of which three contained more than two species) and five more clades in the chloroplast analysis (one with more than two species). None of these clades where contradicted (with a pp. equal to, or greater than 0.95) in the other analysis and only the subclades containing at least three taxa will be discussed further. 

It is worth noting that the two clades including *P. villosa* - *P. pulvinaris* and *P. chinensis* - *P. discolor* were found in both phylogenies. The nuclear analysis revealed one clade including *Potentilla crantzii* (Crantz) Fritsch, *P. recta* L., *P. thuringiaca* Bernh. ex Link, *P. tanacetifolia* Willd. ex Schlecht., *P. kurdica* Boiss. & Hohen.*, P. chrysantha* Trevir.*, P. caucasica* Juz.*, P. tabernaemontani* Asch. and *P. neumanniana *Rchb. A subclade spanning *P. tanacetifolia - P. neumanniana*, also had a pp. of 1. Two morphological subgroups are found in this clade: upright, pinnate–digitate species (*P. crantzii*, *P. recta*, *P. thuringiaca* and *P. tanacetifolia*), and decumbent, trifoliate–digitate species (*P. kurdica, P. chrysantha, P. caucasica, P. tabernaemontani* and *P. neumanniana*) (Fig. 4). Another strongly supported clade in the nuclear analysis (pp. 1) contained *P. multifida* L.*, P. pedersenii* Rydb.and *P. rubricaulis* Lehm. These three species are morphologically coherent with basically pinnate leaves and crispate wooly hairs on the lower leaf surface [66]. The analysis of the chloroplast data resulted in a subclade including *P. uniflora* Ledeb.*, P. subvahliana* Jurtzev*, P. gorodkovii* Jurtzev*, P. villosa* Pall. ex Pursh, and *P. pulvinaris* Fenzl. *Potentilla villosa* and *P. pulvinaris* also formed a clade with pp. 1 in the nuclear analysis. Species in the clade are morphologically coherent as most of them are cushion-forming, trifoliate and have large flowers on unbranched or sparsely branched inflorescences. The lower side of the leaves are covered by crispate wooly hairs. The dominating style type in the clade is Conostylae, but five out of the 37 taxa investigated had Gomphostylae type of styles (Table S1 and Fig. 5).

Five of the six clades discussed above (i.e. not including the Anserina clade) are further joined in three clades with strong support in both phylogenies. 1) A clade containing the ivesioid and Argentea clades (E and F) had pp. 1 in the nuclear tree and pp. 0.95 in the chloroplast tree. 2) A clade containing the Fragarioides, Reptans, ivesioid and Argentea clades (C, D, E and F) had pp. 1 and pp.0.94 in the chloroplast and nuclear trees, respectively and 3) a clade containing Alba, Fragarioides, Reptans, ivesioid and Argentea clades (B, C, D, E and F) had pp. 1 in both trees.

*Ancestral state reconstruction* – The likelihood-ratio test favored the SYN model for both the style- and anther character (p=0.002 in both cases). The scaled likelihoods of each ancestral state, and for a selected number of nodes, are illustrated in figures 5 and 6.

**Figure:**
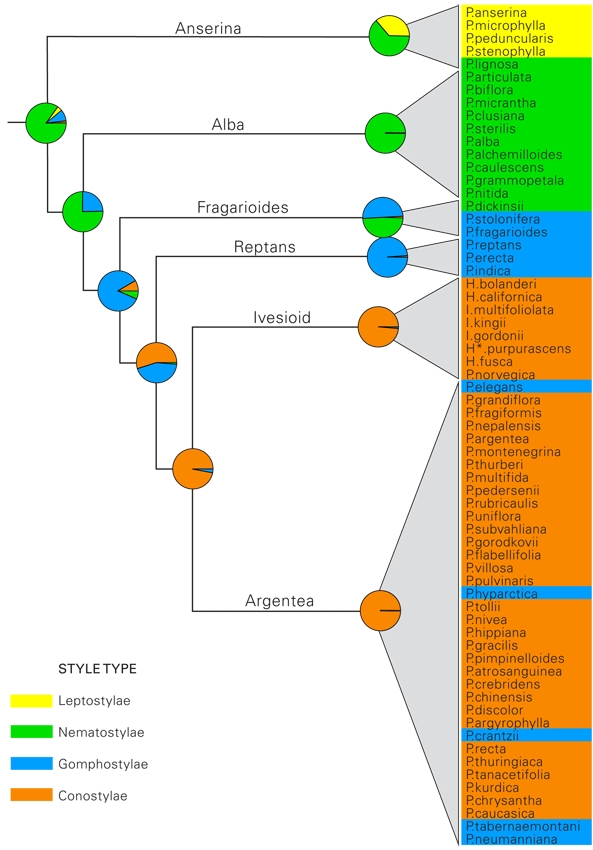
**Figure 5.** Ancestral state reconstruction of the style types identified by Wolf [4] indicated on a simplified tree from figure 4. Species are color-coded according to the style types defined by Wolf [4]. Yellow, indicates the short threadlike Leptostylae type (fig. 1d), green; the long and threadlike Nematostylae type (fig. 1a), blue; the club-shaped Gomphostylae type (fig. 1c) and orange; the cone-shaped Conostylae type (fig. 1b). Species names have been abbreviated as follows: P, *Potentilla*; H, *Horkelia*; I, *Ivesia* and H*, *Horkeliella*.

**Figure:**
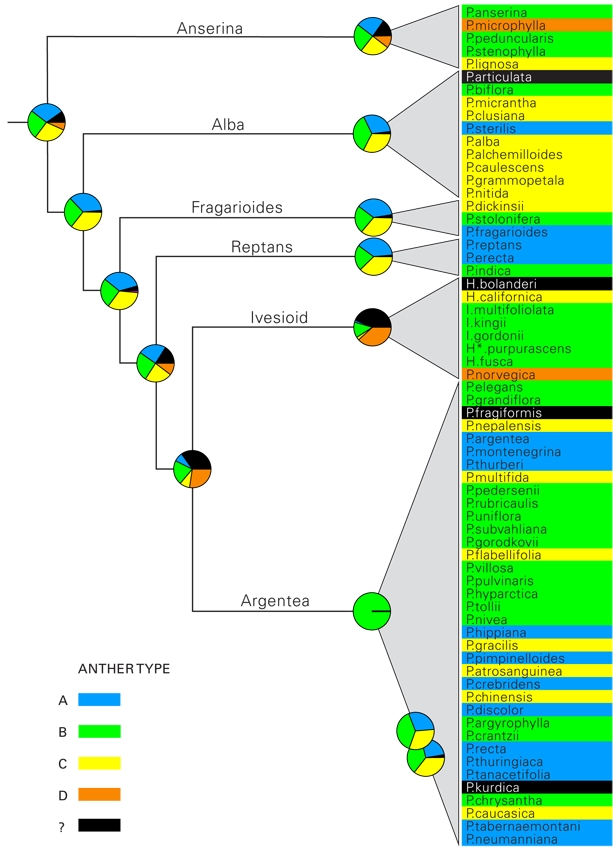
**Figure 6. **Ancestral state reconstruction of the anther types identified in this investigation, indicated on a simplified tree from figure 4. Species are color-coded according to the identified style types and corresponds to the letters A-D in figure 2. Voucher material for four taxa was of too poor quality, or not available at the time of the analysis and is represented by a question mark. Species names have been abbreviated as in figure 5.

## Discussion

 The Anserina clade – This clade is sister to the rest of *Potentilla* in the chloroplast analysis (Fig. 3), but there was weak support (pp. 0.61) for a sister relationship to the outgroup Fragariinae in the nuclear analysis (Fig. 4). The support for a Fragariinae-Anserina clade increased slightly to pp. 0.84 in an analysis of the same matrix excluding the indel data (results not shown). An unresolved position for the Anserina clade was observed by Lundberg et al. [19] who used a much broader sample from the Fragariinae clade, but only *P. lignosa* as a representative of the Anserina clade. The analyses by Eriksson et al. [2] [3] gave the same result when the Anserina clade was represented by *P. anserina*, *P. peduncularis* and *P. stenophylla*. Hence, low-copy nuclear markers may be needed to resolve the nuclear origin of this clade. The position of *P. stenophylla* and *P. microphylla* relative to *P. peduncularis* differs between the two phylogenies which might be due to hybridization. However, a large part of the nuclear *P. stenophylla* sequence is missing from the analyzed matrix, so analytical errors cannot be ruled out. *Potentilla lignosa* has a strongly supported position (pp. 1) as sister to the rest of the Anserina clade in both analyses. Wolf [4] placed *P. lignosa* in the group Xylorrhizae together with *P. lindenbergii* Lehm., a synonym for *Sibbaldianthe adpressa* Bunge, but Lundberg et al. [19] recently showed that *S. adpressa* is not closely related to *P. lignosa*. Furthermore, Botschantzev [67] transferred *P. lignosa* to the genus *Tylosperma* Leeuwenb. and Soják [5] recently added* P. sericophylla* Parker to the same genus based on structures of the anthers. It is worth noting that we have found no support for a phylogenetic signal in anther morphology in the species treated in this study (Fig. 6). *Potentilla anserina* and related species (that are not included in this study) have been proposed to be transferred to the genus *Argentina* Lam. [68]. Our phylogenetic results confirm that a transfer of the species in the Anserina clade to *Argentina* would not lead to any taxonomic consequences for the remainder of *Potentilla*, which has a sister relationship to the Argentina clade. However, unless support for a sister relationship to Fragariinae will increase with the addition of more data, it may be more reasonable to keep the species of the Anserina clade classified in *Potentilla* in order to minimize name changes. 

The Alba clade - The majority of species in the Alba clade (*P. alba, P. sterilis, P. nitida, P. alchemilloides, P. grammopetala *and* P. caulescens*) have white to pink petals and a European distribution. *Potentilla sterilis* and *P. alba* occur in the lowland of northern Europe and a close relationship is supported by both chloroplast and nuclear data. *Potentilla micrantha* has a wide distribution similar to that of *P. sterilis*, but occurs at higher altitudes and further to the east. The remaining taxa of the core group are more or less narrow endemics of different European mountain ranges. The distribution of *P. micrantha* overlaps the alpine species in some places, although they do not occur at the same elevation. In addition, the two yellow-flowered species *P. biflora* and *P. articulata* are part of the Alba clade. The distribution of *P. biflora* spans from the Caucasus, across China to the Siberian and North American arctic, while *P. articulata* has a more restricted Himalayan distribution. The position of *P. articulata* differs between the analyses. In the nuclear tree, it joins *P. biflora* as sister to the rest of the clade (pp. 1), while in the plastid tree it joins the rest of the Alba clade (pp. 0.98) to leave *P. biflora* as single sister taxon to the rest of the Alba clade. As little is know about ploidal levels for *P. articulata* and we have no additional data, the reason for this incongruence remains unknown.

The Fragarioides clade – *Potentilla fragarioides* and *P. stolonifera* are very similar in their morphology, and sometimes difficult to tell apart. Ledebour [69] notes in the original description of *P. stolonifera* that it differs from *P. fragarioides* by having stolons and more narrow and obtuse terminal leaflets. He also mentions a geographical, east-west distinction between the two. In the nuclear phylogeny the two taxa form a clade with *P. dickinsii*, making a group of plants with pinnate leaves and an Asian-Pacific distribution. The *P. fragarioides *and* P. stolonifera* clade is also present in the chloroplast analysis but not including *P. dickinsii. *The phylogenetic position of this clade, relative the Reptans clade, differs between the two analyses. Only diploids are reported from *P. dickinsii* and *P. fragarioides* (the ploidal level of *P. stolonifera* is unknown) and because of this, allopolyploidy is not a plausible explanation for the evolution of species in the clade. However, additional sampling of Asian species might help with the interpretation of this incongruence (as well as the one discussed below). Dobes and Pauli [10] included *P. fragarioides* in their analysis and showed that it forms a clade with *P. freyniana *Bornm (pp. 1). that excludes *P. dickinsii* (pp. 0.9)*,* and hence, corresponds to the Fragarioides clade in our analysis. In their analysis, this clade is sister to the Argentea clade, which differs from our result. Hence, we believe more analyses are needed to resolve the phylogenetic origin of the Fragarioides clade.

The Reptans clade - This clade includes *P. reptans* (type species of genus *Potentilla*), *P. indica* and *P. erecta* and corresponds to the group Tormentillae proposed by Wolf [4]. Its position in the two analyses is shifting in relation to the Fragarioides clade. Hybridization is a plausible explanation for this incongruence, as polyploids have been reported from all three species in the Reptans clade. In fact, *P. indica* is the only species outside of the Argentea clade from where 2n>10x have been reported (but see the discussion about *P. norvegica* below). *Potentilla reptans* and *P. indica* both have stolons like *P. stolonifera* from the Fragarioides clade, but the stolons differ in their morphology [4]. *Potentilla erecta* is one of few species in the genus with four instead of five petals. Furthermore, *Potentilla indica *is distiguished by having a swollen strawberry-like receptacle at fruit maturity. The species was previously placed in the genus *Duschesnea,* but both morphology as well as molecular data in both this and previous studies [2] [3] [4] unambiguously place this species in *Potentilla*.

The ivesioid clade - The clade comprises plants with a distribution restricted to the western United States and northern Baja California [70]. Several species, especially from the genus Ivesia (comprising ~30 species [71]) have conspicuous morphological traits interpreted as adaptations to drought. These include small flowers with threadlike petals and small curved leaflets that are densely covered with hairs. Still, morphological diversity is large in this clade. The genus Horkelia (~20 species [71]) is morphologically a much more coherent group than Ivesia, but with less distinct species boundaries. One character used to distinguish Horkelia from Ivesia, and related species, is a deep hypanthium carrying flat upright stamens, a character also shared with European species in the Alba clade, for example *P. caulescens. *Sister to this clade of narrowly distributed taxa is the widespread species *P. norvegica* (nuclear analysis, Fig. 4) that shifts position to the Argentea clade in the chloroplast analysis (Fig. 3). *Potentilla norvegica* (octaploid – dodecaploid) is a short-lived ruderal herb and the only representative in this study from the large, but morphologically coherent Rivales group [4]. This incongruence is likely to be caused by hybridization, given the high ploidal levels (2n=8x, 10x) found in *P. norvegica*. The clade has a well-supported position inside *Potentilla *and the included taxa have all been proposed to belong to genus *Potentilla* at some time in the past (cf. [2]). A full taxonomic revision of *Potentilla*, based on molecular data as well as morphology, would be useful in order to resolve the issue of genera and species names in this part of the Potentilleae clade. It seems reasonable that generic classifications attempts to follow phylogeny, i.e. genera should be monophyletic. Given this position, it is not possible to maintain the separate genera of the ivesioid clade, as this would cause *Potentilla* to be paraphyletic. One plausible solution to this problem is to classify all ivesioid species as *Potentilla *and give the clade subgeneric rank. Another option, if generic status is requested for reasons of distinctness, is to divide *Potentilla* into several genera reflecting the major clades found in this study. Some generic names for these clades are already available as taxonomists have previously argued for splitting *Potentilla*. Examples of such relevant generic names are *Argentina* Lam. (the Anserina clade) and *Fragariastrum* Heist. (the Alba clade). However, we question the usefulness of the latter exercise, considering the great number of species that would need to be transferred from *Potentilla*, and the subsequent name changes that would be required. Furthermore, although the clades identified in *Potentilla* are well supported by sequence data, several of them are currently difficult to distinguish morphologically. 

The Argentea clade - This clade comprises more than half of the species included in this study. Many of the species have several ploidal levels reported and taxa with levels between octo- and dodecaploid are with few exceptions only reported from the Argentea clade. One of the exceptions is *P. indica* (2n=10x, 12x) from the Reptans clade, the other is *P. norvegica* that has its chloroplast origin in the Argentea clade but in the nuclear analysis is sister to the ivesioid clade. It is possible that more species with high ploidal levels will be found outside the Argentea clade when more taxa are added to the dataset. Still, it is clear that high ploidal levels are overrepresented in this clade (Table S1). Resolution within the Argentea clade was generally low and only four subclades with good support (pp. >0.95) including three or more species were found (Fig. 3 & 4). In addition, eight more clades with good support and including two taxa each were found in the analysis. Since none of these twelve clades are contradicted, and given the method used in this study, no evidence for allopolyploidization has been found in the Argentea clade despite its abundance of high ploidal level species.

## Possible hybridization events

Polyploidy is a widespread phenomenon in the genus *Potentilla*. Ploidal levels from 2x up to 13x have been reported from the about 80 species for which chromosome counts have been performed (Table S1). Diploids constitute approximately one third of these. However, there seems to exist an array of ploidal levels within many species and 2x is rarely reported as the only chromosome number for a taxon. Hybridization and allopolyploid speciation may be the process behind this wealth of ploidal levels. Taxonomically, these scenarios could lead to problems with species delimitation or that a hybrid swarm of taxa would be considered one single variable species. This could be the explanation for the multitude of taxa in *Potentilla* that are difficult to delimit taxonomically [14] [15] [72] and the vast number of species-, subspecies- and variety names in the genus. A query for the name *Potentilla* on the International Plant Name Index (IPNI) web server [33] in April 2011, resulted in 3163 records of infrageneric and infraspecific names. This can be compared with the about 300 species and 550 infraspecific taxa that Wolf [4] recognized in his revision of *Potentilla*.

Allopolyploidy is a plausible explanation for three of the five supported incongruences (pp. ≥0.98) we have identified in *Potentilla*: 1) *Potentilla stenophylla *and *P. microphylla *switch positions in relation to *P. peduncularis *within the Anserina clade. 2) The Reptans and Fragarioides clades switch positions. The Reptans clade is sister to the ivesioid and Argentea clades in the nuclear phylogeny while sister to a clade containing *P. dickinsii*, Fragarioides-, ivesioid- and Argentea clades in the chloroplast tree. 3) *P. norvegica *is part of the Argentea clade in the chloroplast tree while sister to the ivesioid clade in the nuclear tree. The remaining two incongruences involve no polyploid taxa and we believe that other explanations than allopolyploidization are more plausible for them: 4) Within the Alba clade, *P. articulata *(unknown ploidal level) is sister to *P. biflora *(diploid) in the nuclear phylogeny but form a clade with *P. micrantha *in the chloroplast phylogeny. 5) *P. dickinsii *changed position from inside the Fragarioides clade, in the nuclear phylogeny, to a sister position to the Fragarioides, ivesioid and Argentea clades in the chloroplast analysis.

 One incongruence was also found in the outgroup. *Dasiphora fruticosa* splits off basally in the Fragariinae clade in the nuclear tree but is sister to *Drymocallis rupestris* in the chloroplast phylogeny. This result was previously reported by Lundberg et al. [19].

## Systematically informative morphological traits

Figure 5 shows the nuclear phylogenetic relationships of the groups recognized in this study, with style types corresponding to Wolf's [4] definitions highlighted. Also indicated, are the inferred ancestral style character states for a selection of nodes. The slender Leptostylae-type style (Fig. 1d) is unique to the core taxa in the Anserina group. *Potentilla lignosa*, which is morphologically different from the core group, shares its style type with the Alba clade, in which the threadlike Nematostylae-type style (Fig. 1a) is found in all taxa. The majority of species in the Fragarioides and Reptans clades have club-shaped Gomphostylae-type styles (Fig. 1c), except for *P. dickinsii* which has a style of the Nematostylae type (Fig. 1a). The Argentea clade is dominated by taxa having a conical Conostylae-type style (Fig. 1b), but some admixture of Gomphostylae-type styles (Fig. 1c) does occur.

 Species from the ivesioid clade were never included in Wolf's [4] revision of *Potentilla, *and therefore never subject to the classification based on style types. Still, their styles are very distinct and resemble the Conostylae type with a broad base tapering to the apex and then abruptly turning into a stigma (Fig. 1b). However, the styles of many species are long and in many aspects approaching the filiform Nematostylae type (Fig. 1a). Many species in the ivesioid clade are adapted to bee pollination and have deep hypanthia and often erect stamens forming a tube. This type of flower morphology is rare in *Potentilla*, but can also be found in the European species *P. caulescens *and* P. grammopetala* from the Alba clade, and three similar species not included in this study. We hypothesize that the long styles among ivesioid species have co-evolved with other morphological features of the flower to conform to this pollination syndrome and that the style type in fact has a Conostylae type origin.

A number of anther types may also be recognized within the genus (Table S1). The anthers vary in the size of the connective compared to the thecae as well as in where the filament is attached (Fig. 2). However, a classification of anther types into a number of categories, and subsequent optimization of these characters on the phylogeny, does not seem to reflect phylogenetic relationships as is evident by the ancestral state reconstruction presented in Figure 6. One exception seems to be the most recent common ancestor (MRCA) state of the Argentea clade that is inferred to be character state B (Fig. 2b and 6). This result is interpreted to be an effect of the method used for the character optimization. The method requires a fully resolved tree, which can be achieved by adding zero length branches to the phylogeny before performing the ancestral state reconstruction. The order of the nodes in the resolved clade, and hence the inferred ancestral states, are therefore highly influenced by the process of creating the dichotomous tree. A more reliable result is the inferred states of the two sub-clades with pp. 1 (spanning *P. crantzii – P. neumanniana* and *P. tanacetifolia – P. neumanniana,* respectively), which show approximately equal support for the MRCA character states A, B and C for these clades. Hence, the overall pattern in our analysis show that style type is a good indicator of phylogenetic relationships in *Potentilla, *whereas anther morphology (as defined here) is a homoplastic character and not useful for studying natural groups in *Potentilla* as a whole.

## Conclusions

Six major clades within genus *Potentilla* were identified and informally named. These clades have good support in both the chloroplast and the nuclear phylogenies and, except for the Fragarioides clade, correspond well with the results of Dobes and Paule [10] who used an extensive chloroplast dataset and a taxon sample that partially overlap ours. Furthermore, this study has identified four well-supported incongruences in the genus *Potentilla*, of which four include polyploid taxa, and are considered candidates for further investigations of allopolyploid origin. Taxa from the proposed segregate genera *Horkelia, Ivesia, Horkeliella* (here treated as one group, the ivesioids) and *Argentina* form monophyletic groups, respectively. The ivesioid clade is nested within the *Potentilla* clade*,* which makes the genus *Potentilla* paraphyletic if segregate genera are recognized. We tentatively suggest that these species should be classified in *Potentilla*. Style morphology, in contrast to anther shape, is a morphological character that seems to reflect the phylogenetic relationship between species in the *Potentilla* clade. Therefore, taxonomic classification relying on anther shape must be considered unsettled until corroborated by molecular data.

## Acknowledgements

This study was funded by the Swedish Research Council, grant 2004-1698. We acknowledge Anne-Cathrine Sheen and Bernard Pfiel for valuable comments on the manuscript. We also like to thank the Gothenburg Botanical garden for providing plant material and for taking care of the seedlings until they flowered. Further, we thank Katarina Karlsson for the translation of parts of Theodor Wolf's work, and Barbara Ertter for providing a preview of the ongoing work on *Potentilla* and related genera in Flora of North America.

## Competing interests 

The authors have declared that no competing interests exist.

## Supplementary information

Phylogenetic trees and alignments have been deposited at Dryad: doi:10.5061/dryad.8f3bk

****

**Supplementary Table 1.** Species included in the investigation with reported ploidal levels, clade affinity and the sequences used in the phylogenetic analysis indicated. Character states from the morphological analyses are explained in figure 1 (style shape) and figure 2 (anther shape). Vouchers pertain to the sequences but was also used for the morphological analysis. 

**Table:** 

**Taxa**	**Ploidal level(s)**	**Clade**	**Style shape**	**Anther shape**	**Voucher**	**GenBank accession numbers**	** **	** **	** **
** **	** **	** **	** **	** **	** **	**ITS**	**ETS**	**TrnL/F**	**TrnS/G**
Dasiphora fruticosa		Outgroup			See [19]	U90808[2]/U90809[2]	FJ422355[19]	AJ512233[3]	FJ422316[19]
Fragaria vesca		Outgroup			Eriksson & Smedmark 43 (S)	AJ511771[3]	FJ422362[19]	AJ512232[3]	FJ422324[19]
Rosa majalis		Outgroup			T. Eriksson 641 (GH, S)	U90801[2]	FJ422371[19]	AJ512229[3]	FJ422333[19]
Sanguisorba officinalis		Outgroup			See [19]	U90797[2]	FJ422372[19]	AJ416465[3]	FJ422334[19]
Comarum palustre		Outgroup	Nematostylae		See [19]	FJ356158[19]	FJ422352[19]	AJ512237[3]	FJ422313[19]
Sibbaldia cuneata		Outgroup			Binns 5 (E)	FJ356173[19]	FJ422373[19]	FJ422301[19]	FJ422335[19]
Chamaerhodos mongholica		Outgroup			E. Rosenius 1028 (S)	FJ356155[19]	FJ422349[19]	FJ422285[19]	FJ422312[19]
Potentilla anserina	4x, 5x, 6x [73] [74]	A Anserina	Leptostylae	B	See [3]	FN430824	FN421405	FN561752	FN556670
Potentilla microphylla	2x, 4x [73]	A	L	D	MA 144 (GB)	FN430809	FN421388	FN556412	FN556679
Potentilla peduncularis	4x [73]	A	L	B	MA 173 (GB)	FN430820	FN421389	FN561742	FN594721
Potentilla stenophylla		A	L	B	KGB 299 (GB)	FN555607	FN421381	FN561738	FN556662
Potentilla lignosa		A	N	C	MA 132 (GB)	FJ356171[19]	FJ422369[19]	FJ422299[19]	FJ422332[19]
Potentilla articulata		B Alba	N		KGB 324 (GB)	FN555611	FN421410	FN666414	
Potentilla biflora	2x [73]	B	N	B	Viereck 5042 (S)	FN430826		FN561749	FN556673
Potentilla alba	4x [73]	B	N	C	MA 122 (GB)	FN430774	FN421355	FN556397	FN556667
Potentilla alchemilloides	2x [74]	B	N	C	A. & A.-L. Anderberg 26 (S)	FJ356168[19]	FJ422367[19]	FJ422297[19]	FJ422329[19]
Potentilla caulescens	2x, 6x [73] [74]	B	N	C	MA 133 (GB)	FN430819	FN421379	FN556399	FN594714
Potentilla clusiana	2x, 6x [73] [74]	B	N	C	AA 353 (GB)	FN430812	FN421403	FN556401	FN594711
Potentilla grammopetala		B	N	C	MA 147 (GB)	FN430827	FN421397		FN556671
Potentilla micrantha	2x [73] [74]	B	N	C	Eriksson & Smedmark 42 (SBT), See [3]	FN430823		FN561746	FN556678
Potentilla nitida	6x [74]	B	N	C	TE 825 (S)	FN430795	FN421375	FN561733	FN556663
Potentilla sterilis	4x [73] [74]	B	N	A	TE 734 (S)	FN555612	FN421376	FN561732	FN556655
Potentilla dickinsii	2x [73]	C Fragarioides	N	C	MA 123 (GB)	FN430775	FN421402	FN561727	FN556668
Potentilla fragarioides	2x [73]	C	Gomphostylae	A	Cult. in Hortus Bergianus (seed from China, Bejing). No voucher.	FN555610		FN561747	FN557007
Potentilla stolonifera		C	G	B	BE 1382:1 (GB)	FN430814	FN421363	FN556420	FN556654
Potentilla erecta	2x, 3x, 4x, 5x, aneuploids [73] [74]	D Reptans	G	A	MA 124 (GB)	FN430780		FN556405	FN594699
Potentilla indica	10x, 12x [74]	D	G	B	MA 178 (GB)	FN430828		AJ512242[3]	
Potentilla reptans	4x [73] [74]	D	G	A	MA 131 (GB)	FN430815	FN421368	FN561728	FN556657
Comarella multifoliolata		E ivesioid	Conostylae	B	Eriksson 820 (SBT)	FN430788	FN421373	FN556394	FN594713
Horkelia bolanderi		E	C		Eriksson s.n. (SBT)	FN430789	FN421401	FN556395	FN556664
Horkelia californica	4x [71]	E	C	C	Lewis S. Rose 66086 (GB)		FN421411	FN561751	
Horkelia fusca	4x [71]	E	C	B	Bartholome & Andersson 4901 (GH)	U90795[2]		AJ512247[3]	
Horkeliella purpurascens		E	C	B	Eriksson 818 (SBT)	FN430798	FN421382	FN561739	FN556665
Ivesia gordonii		E	C	B	Higgins & Goodrich 14745 (GH)	U90796[2]		AJ512221[3]	
Ivesia kingii		E	C	B	J. L. Reveal et al. #4782 (GB)	FN430787	FN421377	FN561735	FN556666
Potentilla norvegica	8x, 10x [73] [74]	F Argentea	C	D	BE 1567:1 (GB)	FN430817	FN421362	FN561730	FN594706
Potentilla argentea	2x, 4x, 5x, 6x, 8x, 12x? [73] [74]	F	C	A	MA 143 (GB)	FN430808	FN421387	FN561750	FN594719
Potentilla nepalensis		F	C	C	MA 163 (GB)	FN430821	FN421390	FN561743	FN556672
Potentilla argyrophylla		F	C	B	MA 130 (GB)	FN430822	FN421391	FN561744	FN594720
Potentilla atrosanguinea		F	C	C	MA 125 (GB)	FN430778	FN421372	FN556398	FN556658
Potentilla caucasica		F	C	C	AA 346 (GB)	FN430802	FN421378	FN561736	FN594700
Potentilla chrysantha	4x, 6x, 8x [74]	F	C	B	MA 142 (GB)	FN430803	FN421385	FN556400	FN556653
Potentilla kurdica		F	C		MA 140 (GB)	FN430813	FN421395	FN556411	FN556669
Potentilla pimpinelloides	2x, 10x [74]	F	C	A	MA 139 (GB)	FN430793	FN421384	FN556417	FN594716
Potentilla recta	2x, 4x, 5x, 6x, 8x [73] [74]	F	C	A	Eriksson s.n. (SBT)	FN430784	FN421393	FN556419	FN594702
Potentilla tanacetifolia	4x [73]	F	C	A	Eriksson s.n. (SBT)	FN430797	FN421366	FN556422	FN594717
Potentilla thuringiaca	6x, 8x [73] [74]	F	C	A	MA 119 (GB)	FN430777	FN421406	FN556423	FN594698
Potentilla crantzii	4x, 5x, 6x, 7x, aneuploids [73] [74]	F	G	B	TE 703 (S)	FN555609		FN556402	FN556659
Potentilla neumanniana	2x, 4x	F	G	A	Cult. Hortus Bergianus, no voucher.	FN666607	FN421370	FN556414	FN556652
Potentilla tabernaemontani	4x, 5x, 6x, 7x, 8x, 9x, 10x, 12x, aneuploids [73] [74]	F	G	A	Voucher missing	FN555608	FN421365	FN556466	FN594703
Potentilla hyparctica	6x [73] [74]	F	G	B	BE 208-1-05 (GB)	FN430781	FN421360	FN556410	FN594709
Potentilla chinensis	2x [73]	F	C	C	Eriksson s.n. (SBT)	FN430825		FN561748	FN594718
Potentilla crebridens	4x [73]	F	C	A	BE 569:4 (GB)	FN430811	FN421356	FN561731	FN556674
Potentilla discolor		F	C	A	MA 141 (GB)	FN430794	FN421396		FN556675
Potentilla gracilis	12x, 13x [73]	F	C	C	MA 146 (GB)	FN430805	FN421399	FN556464	FN557006
Potentilla hippiana	12x [73]	F	C	A	Eriksson & Smedmark 28 (SBT) (ex. RBG Edinburgh, accession 1962.0795)	FN430801	FN421359	FN556409	
Potentilla nivea	2x, 3x, 4x, 5x, 6x, 7x, 8x, 10x [73] [74]	F	C	B	BE 1672:1 (GB)	FN430816	FN421371	FN561729	FN594705
Potentilla tollii		F	C	B	MA 145 (GB)	FN430807	FN421407	FN556424	
Potentilla flabellifolia		F	C	C	MA 164 (GB)	FN430810	FN421392	FN556406	FN556677
Potentilla gorodkovii		F	C	B	BE 930-05-1 (GB)	FN430800	FN421380	FN556408	FN556661
Potentilla pulvinaris		F	C	B	MA 134 (GB)	FN430791	FN421394	FN556418	FN594701
Potentilla subvahliana	4x [71]	F	C	B	BE 931-1-5 (GB)	FN430783	FN421364	FN556421	FN594707
Potentilla uniflora	2x, 3x, 4x, 6x [73]	F	C	B	BE 271-4-05 (GB)	FN430785	FN421367	FN556425	FN594708
Potentilla villosa	2x [73]	F	C	B	MA 127 (GB)	FN430786	FN421369	FN556426	FN594704
Potentilla fragiformis	6x, 8x	F	C		BE 540-1-05 (GB)	FN430790	FN421386	FN556407	FN594710
Potentilla grandiflora	(2x), 4x, (6x)	F	C	B	MA 149 (GB)	FN430806	FN421400	FN556465	
Potentilla montenegrina	4x, 6x	F	C	A	Eriksson & Smedmark 30 (SBT) (ex. RBG Edinburgh, accession 1962.1848A)	FN430782	FN421361	FN556413	FN556651
Potentilla multifida	(2x), 4x, (6x)	F	C	C	TE 705 (S)	FN430818	FN421374	FN561734	FN594712
Potentilla pedersenii		F	C	B	BE 05/24 (GB)	FN430799	FN421404	FN556415	FN556660
Potentilla rubricaulis	8x? [71]	F	C	B	Nils Alroth 29/7 1960 (GB)		FN421412	FN561745	
Potentilla thurberi		F	C	A	MA 138 (GB)	FN430792	FN421383	FN561740	FN594715
Potentilla elegans	2x, 4x [71]	F	G	B	BE 1440:1 (GB)	FN430779	FN421358	FN556404	FN556676
